# Assessing the association between triglyceride-glucose index and atrial fibrillation: a systematic review and meta-analysis

**DOI:** 10.1186/s40001-024-01716-8

**Published:** 2024-02-12

**Authors:** Alireza Azarboo, Amir Hossein Behnoush, Zahra Vaziri, Mohammad Shahabaddin Daneshvar, Aryan Taghvaei, Arash Jalali, Alessandro Cannavo, Amirmohammad Khalaji

**Affiliations:** 1 Cardiovascular Diseases Research Institute, Tehran Heart Center, Tehran University of Medical Sciences, Tehran, Iran; 2 School of Medicine, Tehran University of Medical Sciences, Poursina St., Keshavarz Blvd., Tehran, 1417613151 Iran; 3 Student Research Committee, Babol University of Medical Sciences, Babol, Iran; 4 Department of Epidemiology and Biostatistics, School of Public Health, Tehran University of Medical Sciences, Tehran, Iran; 5 Department of Translational Medical Sciences, Federico II University of Naples, Naples, Italy

**Keywords:** Atrial fibrillation, Triglyceride-glucose index, Insulin resistance, Systematic review, Meta-analysis

## Abstract

**Background:**

An essential relationship between insulin resistance (IR) and atrial fibrillation (AF) has been demonstrated. Among the methods used to assess IR, the triglyceride-glucose (TyG) index is the more straightforward, dimensionless, and low-cost tool. However, the possible usage of this index in clinical practice to predict and diagnose AF has yet to be determined and consolidated.

**Objective and rationale:**

Herein, we performed a systematic review and meta-analysis to assess the association between the TyG index and AF.

**Methods:**

Databases (PubMed, Embase, Scopus, and Web of Science) were systematically searched for studies evaluating the TyG index in AF. The inclusion criteria were observational studies investigating AF and TyG index correlation in individuals older than 18 years, while preclinical studies and those without the relevant data were excluded. Random effect meta-analyses comparing TyG levels between AF and non-AF cases, AF recurrence after radiofrequency ablation, and post-procedural AF were performed using standardized mean differences (SMD) with their matching 95% confidence intervals (CIs).

**Results:**

Our screening identified nine studies to be analyzed, including 6,171 participants including 886 with AF. The meta-analysis demonstrated that the TyG index resulted higher in patients with AF than non-AF counterparts (SMD 1.23, 95% CI 0.71 to 1.75, *I*^*2*^ 98%,* P* < 0.001). Subgroup analysis showed the same results for post-procedure AF (SMD 0.99, 95% CI 0.78 to 1.20, *I*^*2*^ 10%, *P* < 0.001) and post-ablation AF (SMD 1.25, 95% CI 1.07 to 1.43, *I*^*2*^ 46%, *P* < 0.001), while no difference was found in population-based cohorts (SMD 1.45, 95% CI − 0.41 to 3.31, *I*^*2*^ 100%, *P* = 0.13). Publication year (*P* = 0.036) and sample size (*P* = 0.003) showed significant associations with the effect size, using multivariable meta-regression.

**Conclusion:**

The TyG index is an easy-to-measure surrogate marker of IR in patients with AF. Further clinical studies are warranted to demonstrate its ability for routine clinical use and as a screening tool.

**Supplementary Information:**

The online version contains supplementary material available at 10.1186/s40001-024-01716-8.

## Introduction

As the most prevalent cardiac rhythm disorder, atrial fibrillation (AF) dramatically interferes with patients’ quality of life and is associated with a 10–25% increase in all-cause mortality and more than 8 million disability-adjusted life years [1–3]. AF affects approximately 60 million individuals worldwide, with an incidence that escalates significantly with age as a reflection of comorbidities, cardiovascular risk factors, and metabolic disorders, such as diabetes [1, 4–6]. In this regard, multiple studies have assessed the association between AF and diabetes, with diabetic patients showing an approximate 35% increased risk of developing AF in comparison to nondiabetic ones [7–9]. In line with this data, different reports supported insulin resistance (IR), the main feature of diabetes, as the main factor strictly intertwined with AF pathogenesis [10, 11]. However, aside from diabetes, IR represents a metabolic substrate associated with several cardiovascular disorders, obesity, and inflammation, all risk factors for AF development [12–15]. Indeed, in nondiabetic individuals, studies have provided evidence of a positive correlation between IR and AF risk [16]. For this reason, several tools, like the homeostasis model assessment index for insulin resistance (HOMA-IR), the gold standard for assessing IR, have been used to examine this relationship in the community [17]. Analogously, different studies used a simpler and more low-cost estimating tool called the triglyceride–glucose (TyG) index, which has a power of convenience compared to the HOMA-IR [18]. Moreover, this index has been investigated in several cardiovascular conditions and diseases [18–21].

The TyG index calculation could be performed using the routinely measured parameters, and its application in clinical practice has been widely proposed as it has an enormous diagnostic and prognostic power to assess many IR-related disorders. Based on this premise, we conducted a systematic review and meta-analysis to analyze the data available, establishing the association between the TyG index and AF.

## Methods

This systematic review and meta-analysis was conducted in accordance with the “preferred reporting items for systematic reviews and meta-analyses” (PRISMA) guidelines and was registered in the PROSPERO registry by registration number (CRD42023456875) [22].

### Systematic search strategy

Articles included in this review were identified via comprehensive systematic searches of electronic databases of PubMed, Embase, Scopus, and Web of Science from inception to September 2023. MeSH terms and keywords, including “Atrial Fibrillation” OR “Auricular Fibrillation” OR “Familial Atrial Fibrillation” AND “triglyceride-glucose index” OR “TyG” were combined to elicit original studies on TyG index and AF association. The full search strings applied in each database are included in Additional file 1: Table S1. Two reviewers (SD, AT), using EndNote 9 software (Tomson Reuters, New York, USA), independently evaluated each article, and they also reviewed the full text and eliminated any duplicates. The inclusion–exclusion criteria were followed for selecting studies. The third author (AA) served as the moderator of consensus sessions to resolve any disagreements that might arise between reviewers.

### Inclusion and exclusion criteria

#### Inclusion criteria

The inclusion criteria of the original studies were: (1) studies with an observational study design; (2) studies investigating the AF and the TyG index correlation, in addition to those assessing the effect of the TyG index on incident AF; (3) individuals older than 18 years of age of any ethnicity and both sexes and (4) studies in which electrocardiogram (ECG) findings confirmed AF diagnosis. The TyG index is calculated from fasting plasma glucose (FPG) and triglyceride (TG):

$${\text{TyG}} = \ln \left( {{\text{TG}}\left( {\frac{{{\text{mg}}}}{{{\text{dL}}}}} \right) \times \frac{{{\text{FPG}}\left( {\frac{{{\text{mg}}}}{{{\text{dL}}}}} \right)}}{2}} \right).$$

#### Exclusion criteria

The exclusion criteria were: (1) preclinical studies (in vivo in animals or in vitro in cells); (2) interventional studies, book chapters, reviews, and case reports, (3) studies with incomplete data or an unclear method of the TyG index calculation, and (4) conference abstracts and preprints.

### Data extraction and quality assessment

Two authors (SD, AT), after thoroughly conducting full-text screening, independently input the following information into a pre-piloted, standardized Excel spreadsheet: author and publication year, nationality, design of study, definition of case and control groups, TyG index, sex ratio, age, sample size, and body mass index (BMI). We also extracted the hazard ratio (HR) with the highest number of factors for adjustment to compare the highest TyG index groups to the lowest TyG group. Moreover, when the TyG index was used in analyses as a continuous variable, we extracted the HRs reflecting the risk per one-unit increase of the TyG. We extracted the area under the curve (AUC) from studies that reported AUC for the diagnosis of AF or outcome prediction. The extraction was performed using a sheet in Microsoft Excel 2016.

For evaluation of the qualities of the included studies, the Newcastle–Ottawa Scale (NOS) was employed [23]. Selection, comparability, and outcome are the three main domains for quality assessment of cohort studies. Based on this scale, a score of 3 or 4 in the selection domain, 1 or 2 in the comparability domain, and 2 or 3 in the outcome domain is considered “good” quality. Two independent authors (SD and AT) assessed the qualities, and in cases of disagreement, a third author (AA) resolved the issue.

### Statistical analysis

R version 4.3.0 (R Core Team [2020]. R: A language and environment for statistical computing. R Foundation for Statistical Computing, Vienna, Austria) was used to conduct statistical analysis, and the packages for analyses were “meta” and “metafor”.

In cases of reporting median and interquartile range (IQR) or median and range for any of the variables needed, we calculated mean and standard deviation through the methods suggested by Luo et al. and Wan et al. [24, 25]. To evaluate the relationship between the TyG and the AF incidence, we employed the Hedges’ g standardized mean differences (SMD) with their matching 95% confidence intervals (CIs) by random-effect meta-analysis, as a general indicator [26]. Since the TyG index has a logarithmic scale without any unit, we used mean difference (MD) for the meta-analysis as a sensitivity analysis. The statistical heterogeneity was assessed using the *Q*-test and *I*^*2*^. It was determined that *I*^*2*^ values of 25, 50, and 75%, respectively, indicated minimal, moderate, and high heterogeneity. To assess the publication bias, Egger's test was conducted [27]. Meta-regression based on publication year, male percentage, mean age, and sample size was performed to assess their effect on overall heterogeneity. Subgroup analysis was also performed for different populations, when possible. A two-sided *P* less than 0.05 was used to indicate statistical significance.

## Results

### Study selection and baseline characteristics

**Fig. 1:**
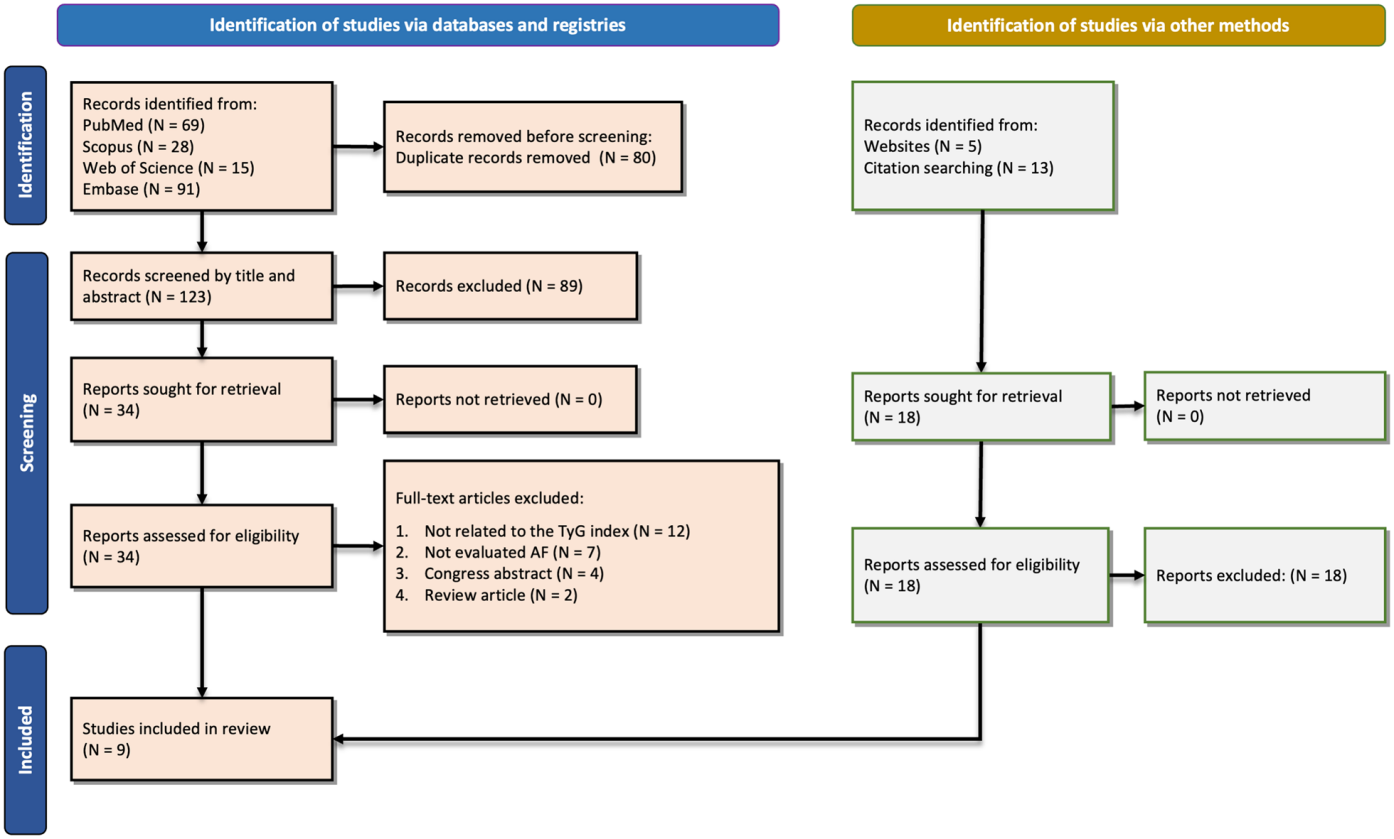
PRISMA flowchart of the study selection process

We identified two hundred and three records from an initial screening of the four databases analyzed and described in the methods. As reported in Fig. 1, 80 studies were immediately removed because they were duplicated. Then, a further 89 studies were excluded by title/abstract screening, and another 25 were excluded during the full-text assessment for reasons mentioned in Fig. 1. At the end of the screening, nine studies fully met the inclusion criteria and were included in the analysis [28–36]. Studies were conducted mostly in China [28, 29, 32–36], followed by the United States [30] and Sweden [31]. The design of the included studies were prospective cohort [30, 31, 33, 34], retrospective cohort [28, 29], case–control [35, 36], and cross-sectional [32]. In these studies, 886 patients with AF were compared against 5285 without AF, with a mean age of 63.46 ± 10.47 years and 56.81 ± 10.54 years, respectively. The male percentage was also 65.46% in the AF population and 58.47% in non-AF cases. Table 1 summarizes each study’s characteristics, including patient groups, population, country, design, sample size, mean age and BMI, sex/gender percentage (male %), left ventricular ejection fraction (LVEF) percentage, TyG index, and main findings. The studies analyzed assessed TyG levels with new-onset AF in population-based cohorts, type 2 diabetes mellitus (T2DM), or CAD patients. Further, some of these studies evaluated the TyG index in AF patients' adverse events. As illustrated in Additional file 1: Table S2, the included studies had NOS 7–8 and “good” quality based on the criteria.

**Table 1:** Main characteristics and main findings of the included studies

Study	Groups	Population	AF definition	Country	Study design	Sample size	Male%	Mean age	LVEF (%)	TyG index	Main findings
Wei et al. [34]	AF	Patients with hypertrophic obstructive cardiomyopathy after septal myectomy	POAF: Presence of AF lasting ≥ 5min or requiring cardioversion with antiarrhythmic drugs	China	Prospective cohort	61	52.5	56.75 ± 12.35	67.07 ± 6.73	7.41 ± 0.67	TyG index was an independent risk factor for POAF in patients undergoing septal myectomy (OR: 4.218, 95% CI 2.381–7.473, *P* < 0.001)
	Non-AF					348	52.0	49.92 ± 10.85	67.41 ± 6.34	6.9 ± 0.55	
Ling et al. [29]	NOAF after PCI	ST-segment elevation myocardial infarction patients after percutaneous coronary intervention	NOAF: AF lasting ≥ 30 s during post-PCI hospitalization that spontaneously reverted to sinus rhythm or responded to drug cardioversion	China	Retrospective cohort	42	71.4	69.5 ± 4.7	49.17 ± 8.21	9.48 ± 0.75	The TyG index was an independent predictor of NOAF [OR: 8.884, 95% CI 1.570–50.265, *P* = 0.014]
	No AF after PCI					507	80.5	63 ± 10	51.35 ± 6.69	8.75 ± 0.64	
Tang et al. [33]	Late AF recurrence	Patients with AF who underwent RFCA	Long-standing persistent AF: persistent AF (lasting more than 7 days) episodes lasting > 12 months	China	Prospective cohort	70	75.7	64.38 ± 8.04	63.66 ± 5.0	9.42 ± 0.6	The pre-ablation TyG index was an independent risk factor for late recurrence of AF after RFCA (HR: 2.015, 95%CI 1.408–4.117; *P* = 0.009)
	Non-late AF recurrence					205	67.3	55.07 ± 8.93	64.20 ± 5.2	8.68 ± 0.7	
Zhang et al. [36]	NAFLD + AF	Patients diagnosed with NAFLD by ultrasound	AF: a ≥ 30-s rhythm of the heart, undiscernible repetitive P-waves, and irregular RR intermittent diagnosis of AF)/self-reporting AF	China	Case control	204	64.7	68.78 ± 11.15	–	9.12 ± 0.53	TyG was an independent risk factor for AF (OR: 4.84, 95%CI 2.98–7.88, *P* < 0.001)
	Only NAFLD					708	60.6	56.25 ± 10.31	–	8.01 ± 0.44	
Chen et al. [28]	AF	Patients from the department of cardiology	AF: ECG findings (absence of consistent P-waves, presence of rapid, irregular f waves with a frequency of 350–600 b.p.m. and an irregular ventricular response)	China	Retrospective observational	179	53.1	68 ± 2.22	–	8.53 ± 0.17	TyG index was associated with AF (OR: 3.065, 95% CI 1.819–5.166, *P* < 0.001) in nondiabetic subjects. However, the TyG index was not associated with AF in the diabetic subjects
	No AF (control)					179	51.4	67 ± 2.22	–	8.36 ± 0.17	
Shi et al. [32]	AF	Diabetic patients	AF: of ≥ 30 s showing heart rhythm with no discernible repeating P-waves and irregular RR intervals was diagnosed of AF/the subject’s self-report of AF history	China	Cross-sectional	213	72.3	55.97 ± 13.25	–	9.51 ± 0.74	There was a linear correlation between the TyG index and the prevalent AF in a diabetic population. In the fully adjusted model, each SD elevation of TyG casts a 40.6% additional risk for prevalent AF
	No AF (control)					3031	53.65	56.21 ± 10.67	–	9.17 ± 0.67	
Zhang et al. [35]	Recurrent AF	Patients who underwent valvular surgery with concurrent Cox-maze ablation	Long-standing persistent AF: persistent AF (lasting more than 7 days) episodes lasting > 12 months	China	Case control	117	71.8	61.7 ± 12.7	50.1 ± 14.8	9.21 ± 0.38	A higher TyG index is associated with an increased risk of AF recurrence after simultaneous radiofrequency ablation maze IV procedures for heart valve surgery (HR: 2.021, 95% CI 1.374–3.245, *P* < 0.001)
	Non-recurrent AF					307	70.4	56.8 ± 13.7	53.4 ± 17.6	8.34 ± 0.72	
Liu et al. [30]	TyG < 8.8	General population without known cardiovascular disease	AF: Fatal AF, AF event at visit 2, 3, 4, or 5 determined by ECG, AF determined by hospital discharge codes	USA	Prospective cohort	7605	40.3	53.61 ± 5.73	–	8.30 ± 0.32	. In multivariable-adjusted analysis, both < 8.80 (aHR:1.15, 95% CI 1.02–1.29) and > 9.20 levels (aHR 1.18, 95% CI 1.03–1.37) of the TyG index were associated with an increased risk of AF compared with the middle TyG index category (8.80–9.20)
	8.8 ≤ TyG ≤ 9.2					2477	49.9	54.65 ± 5.75	–	8.98 ± 0.12	
	9.2 < TyG					1769	54.4	55.08 ± 5.54	–	9.57 ± 0.32	
Muhammad et al. [31]	TyG Q1	General population	–	Sweden	Prospective cohort	8221	50.1	46.41 ± 7.73	–	3.38 to 4.38	No significant association was observed between AF and TyG index [aHR 0.99, 95%CI 0.89–1.11, *P* = 0.142]
	TyG Q2					8232	65	45.69 ± 7.48	–	4.38 to 4.55	
	TyG Q3					8232	72.7	45.24 ± 7.44	–	4.55 to 4.74	
	TyG Q4					8214	82.1	45.26 ± 6.92	–	4.74 to 6.70	

*AF* atrial fibrillation, *TyG* triglyceride-glucose index, *LVEF* left ventricular ejection fraction, *NOAF* new-onset atrial fibrillation, *POAF* post-operative atrial fibrillation, *RFCA* radiofrequency catheter ablation, *PCI* percutaneous coronary intervention, *NAFLD* non-alcoholic fatty liver disease, *ECG* electrocardiogram

### Meta-analysis of TyG levels in patients with AF

**Fig. 2:**
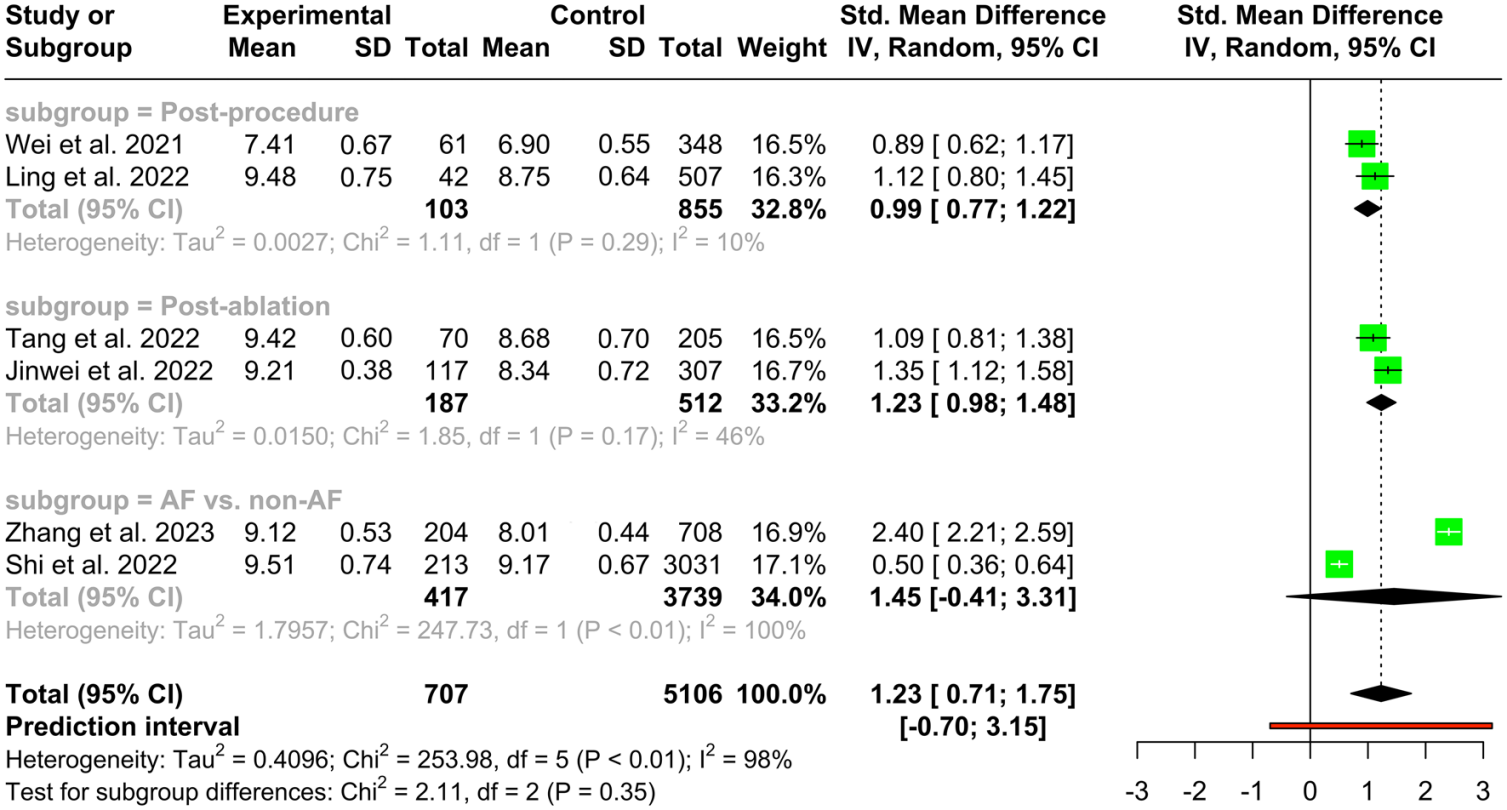
Forest plot and subgroup analysis of the association of mean TyG and AF

Meta-analysis comparing the mean TyG index in patients with and without AF. As shown in Fig. 2, the forest plot, demonstrated a significantly higher TyG index in total AF patients (SMD 1.23, 95% CI 0.71 to 1.75, *P* < 0.001). This analysis was associated with a high heterogeneity (*I*^*2*^: 98%, 95% CI 97.1% to 98.7%). Next, we conducted a subgroup analysis, and as shown in Fig. 2, no significant difference was observed comparing patients with pure AF vs. non-AF controls (SMD 1.45, 95% CI − 0.41 to 3.31, *P* = 0.13). Conversely, as shown in Fig. 2, a higher TyG index was observed in the post-ablation population (SMD 1.25, 95% CI 1.07 to 1.43, *P* < 0.001) that showed late AF recurrence compared to those that did not, and in post-procedural patients with AF (SMD 0.99, 95% CI 0.78 to 1.20, *P* < 0.001) compared to the controls undergoing the same procedures but without AF [septal myectomy or percutaneous coronary intervention (PCI)] (Fig. 2 and Table 1). Sensitivity analysis by leave-one-out method was performed to assess each study’s effect on the overall effect size. The removal of none of the studies led to no change in overall effect size in terms of significance (Additional file 1: Figure S1). Publication bias was assessed by visual inspection of the funnel plots by trim-and-fill method (Additional file 1: Figure S2). No asymmetry was observed in the funnel plot. Similarly, Egger’s test showed no publication bias (*P* = 0.634).

Meta-regression assessing the effect of each variable on overall effect size is summarized in Table 2. Except for publication year which had a significant association with the effect size (*P* = 0.028), the other variables (mean age, sample size, and male %) had no association. Publication year accounted for 44.40% of the heterogeneity observed. The bubble plots for these meta-regressions are shown in Additional file 1: Figures S3–6. While, in the multivariable meta-regression by these variables, shown in Additional file 1: Table S3, sample size and publication year had significant associations with the effect size (*P* = 0.036 and *P* = 0.003), and these variables accounted for 89.01% of the heterogeneity.

**Table 2:** Univariate meta-regression for meta-analysis of TyG index in patients with AF vs. healthy controls

Moderator	No. of studies	Slope	95% CI	*P*-value	*R*^2^ (%)
Publication year	6	0.7637	0.0811 to 1.4462	0.028	44.40
Sample size	6	− 0.0002	− 0.0007 to 0.0003	0.379	0
Mean age	6	0.0485	− 0.1024 to 0.1994	0.528	0
Male %	6	0.0113	− 0.0485 to 0.0710	0.711	0

*CI* confidence interval

Finally, in the sensitivity analysis by using MD instead of SMD, the same significant result of higher TyG index in patients with AF was obtained (MD 0.72, 95% CI 0.49 to 0.94, *P* < 0.001). This analysis had a high heterogeneity (*I*^*2*^: 96.6%, 95% CI 94.6% to 97.9%). The forest plot for this analysis is shown in Additional file 1: Figure S7.

### Association between the TyG and risk of AF

Nine studies investigated the association between the TyG and the incidence of outcomes [28–36]. Table 3 represents the differences in outcomes between groups with low or high TyG index, quartiles (*Q*), and tertiles (*T*) of the TyG, and the TyG as a continuous variable. Generally, AF patients presented with a greater TyG index in most cases and across all populations.

**Table 3:** Risk of AF in different groups/levels of the TyG index

Study	Year	Population	Group 1	Group 2	Group 3	Group 4	Continuous
Population-based cohorts							
Liu et al	2023	Population-based cohort of 15,792 patients aged 45 to 64 from 4 US communities	TyG < 8.8 [aHR 1.15] [95% CI 1.02 to 1.29]*	8.8 < TyG < 9.2 [Ref]	9.2 < TyG [aHR 1.18] [95% CI 1.03 to 1.37]*	–	–
Muhammad et al	2022	General population	Q1 [Ref]	Q2 [aHR 1.02] [95% CI 0.95 to 1.09]	Q3 [aHR 0.97] [95% CI 0.9 to 1.04]	Q4 [aHR 0.96] [95% CI 0.89 to 1.04]	Per 1-unit increase [aHR 0.99] [95% CI 0.89 to 1.11]
Coronary artery disease							
Ling et al	2022	ST-Segment Elevation Myocardial Infarction Patients After Percutaneous Coronary Intervention	–	–	–	–	Per 1-unit increase [OR 8.884] [95% CI 1.57 to 50.265]***
Non-alcoholic fatty liver disease							
Zhang et al	2023	Patients diagnosed with NAFLD by ultrasound	Q1 [Ref]	Q2 [OR 1.2] [95% CI 0.66 to 2.17]	Q3 [OR 1.93] [95% CI 1.07 to 3.49]*	Q4 [OR 4.34] [95% CI 2.37 to 7.94]***	Per 1-unit increase [OR 4.84] [95% CI 2.98 to 7.88]***
Atrial fibrillation recurrence							
Tang et al	2022	Patients with AF who underwent RFCA	–	–	–	–	Per 1-unit increase [HR 2.015] [95% CI 1.408 to 4.117]**

*HR* hazard ratio, *OR* odds ratio, *CI* confidence interval, *NAFLD* non-alcoholic fatty liver disease, *AF* atrial fibrillation, *RFCA* radio frequency catheter ablation

^*^P < 0.05, **P < 0.01, ***P < 0.001

#### Population‑based cohorts

Different studies have evaluated the association between TyG and AF risk in the general population. Liu et al. [30], using data from the Atherosclerosis Risk in Communities (ARIC) study, a U-shaped association was found between the TyG index and the incident AF in the cardiovascular disease-free general population in a 24.2-year follow-up period. In their study, the authors analyzed a population of 11,851 nondiabetic participants who were assigned to groups based on the TyG value tertiles at the baseline (T1: TyG < 8.80; T2: 8.80 ≤ TyG ≤ 9.20; and T3: TyG > 9.20), the analysis revealed that T1 and T3 presented with an incidence rate of 0.69 and 1.13 (per 100 person-years), respectively, and showed an increased risk of AF compared to T2, with an incidence rate of 0.83 (*P* = 0.02). In line with this report, Chen et al. [28] observed that the TyG index was positively associated with AF (OR 2.092, 95% CI 1.412 to 3.100, *P* < 0.001). In particular, the TyG index was higher in nondiabetic patients with AF compared to individuals without AF (OR 3.065, 95% CI 1.819 to 5.166, *P* < 0.001) while no differences were observed in the TyG index between diabetic subjects with or without AF (OR 1.286, 95% CI 0.645 to 2.565, *P* = 0.475). On the other hand, Shi and colleagues [32] demonstrated that the frequency of AF in the diabetes cohort was 6.57%. Every one-SD increase in the TyG value increased the prevalent AF risk by 40.6% in diabetic patients (OR 1.406, 95% CI 1.197 to 1.650, *P* < 0.001). Of note, the higher quartile (Q4) of the TyG index was associated with 2.120 (95% CI 1.37 to 3.29, *P* = 0.002) times greater prevalence odds of AF than Q1.

For their part, Zhang et al. [36] in a population of 912 patients with NAFLD, 204 of them with AF, observed that those with the highest Q of the TyG index had a greater risk of AF incidence (33.3%) compared to those in the lowest Q with 16.7% having AF (OR 4.34, 95% CI 2.37 to 7.94, *P* < 0.001). After adjusting for age, gender, BMI, diabetes, serum creatinine, and total cholesterol), an increase of one unit in the TyG index resulted in a rise in the risk of AF by 4.84 (OR 4.84, 95% CI 2.98 to 7.88, *P* < 0.001). In contrast with these data, Muhammad and coworkers [31] in a general population-based cohort of 32,917 participants (6950 presented with AF) from Sweden and with a follow-up duration of 16.9 years (mean), failed to observe any difference between subjects in the highest *Q* (Q4: 4.74–6.70) of the TyG value compared to individuals in the lowest Q (Q1: 3.38–4.38) [HR 0.96, 95% CI 0.89 to 1.04)] (*P* = 0.14). Additionally, the HR per one-unit increase in the TyG value was the same for cases and controls [HR 0.99, 95% CI 0.89 to 1.11)].

#### Post-procedural AF

Next, we analyzed studies showing the utility of TyG as a post-procedural AF predictor. In this regard, in the study by Ling et al. [29], the authors observed that the patients who underwent PCI and had the higher TyG index had an incidence of 17.8% new-onset AF, while the low TyG group had 3.1% new-onset AF (*P* < 0.001). For their part, Wei et al. [34], according to the cut-off point of 7.60, identified by ROC curve analysis, divided a population of 409 patients (348 had no postoperative (septal myectomy) AF (POAF), and 61 were diagnosed with POAF) into two groups based on the TyG value: low (mean: 6.80 ± 0.44) and high (mean: 7.98 ± 0.37) and found that the patients in the high TyG group had an increased incidence of POAF than those in the low group (45.0% vs. 9.7%, *P* < 0.001).

#### Post-ablation AF recurrence

We also analyzed the data concerning the association between TyG and post-ablation AF recurrence. In this context, in their study, Tang and colleagues [33] examined a total of 275 nondiabetic participants who underwent first-time radiofrequency catheter ablation (RFCA) for AF stratified into three T based on pre-ablation TyG (T1: < 8.67, T2: 8.68–9.37, and T3: ≥ 9.38). Patients in T3 had a higher rate of late AF recurrence compared to participants in the T1 group (54% vs. 12%, *P* < 0.001). In another study, Zhang et al. [35] reported that the HR of higher TyG to be 2.021 (95% CI 1.374 to 3.245, *P* < 0.001), was an effective risk factor for AF recurrence after Cox-maze IV ablation.

### AUC for the prediction of AF and its outcomes

In our last analysis, we examined all the studies showing data regarding the AUC value of TyG. In their study, Chen and coworkers [33], compared the nondiabetic AF patients with those without AF and found that the TyG index displayed an AUC value of 0.600 (95% CI 0.542 to 0.659, *P* = 0.001), an optimal cut-point value of 8.35 with a sensitivity of 65.4%, and a specificity of 52.0%. However, when the TyG index was combined with variables like hypertension and total cholesterol, it showed a higher AUC value of 0.667 (95% CI 0.611 to 0.723, *P* < 0.001), with a cut-off value of 0.466, 71.5% sensitivity, and 58.1% specificity.

Next, Zhang et al. [35] promoted using AUC of TyG to predict AF recurrence after the Cox-maze IV procedure. These authors found an AUC of 0. (95% CI 0.796 to 0.871, *P* < 0.001), supporting the high predictive value for AF recurrence of the TyG in this population of patients. Notably, the cut-off value of the TyG index reported in this study was 8.86, and the sensitivity and specificity were 88.6% and 44.7%, respectively.

In line with this notion, Ling et al. [29] demonstrated that an AUC value of the TyG index of 0.758 (95% CI 0.720 to 0.793, *P* < 0.001) has a predictive value for new-onset AF (NOAF) incidence in ST-segment elevation myocardial infarction (STEMI) patients following PCI.

For their part, Shi et al. [32] showed that the AUC of the TyG index alone for identifying prevalent AF in a diabetic population was 0.631 (95% CI 0.614 to 0.648, *P* < 0.001). In addition, the authors demonstrated that when TyG was added to conventional cardiovascular risk factors, the AUC improved for the detection of prevalent AF (0.825 vs. 0.812, *P* = 0.02), an effect also supported by the continuous net reclassification index (0.227, 95% CI 0.088 to 0.365, *P* = 0.001) and integrated discrimination index (0.007, 95% CI 0.001 to 0.012, *P* = 0.03).

Further, Wei et al. [34] found a moderate predictive value for the TyG index for postoperative AF in hypertrophic obstructive cardiomyopathy patients who underwent septal myectomy, showing an AUC of 0.723 (95% CI 0.650 to 0.796, *P* < 0.001). However, after adding TyG to the model based on conventional risk factors, it only numerically, but not significantly increased the prediction ability of postoperative AF, with an AUC of 0.742 (95% CI 0.671 to 0.814) compared to 0.793 (95% CI 0.726 to 0.860) for the conventional risk factor model alone (*P* = 0.065). The optimal cut-off point for the TyG index was found to be 7.60. At this point, the sensitivity of the TyG index was 44.3%, and the specificity was 90.5%.

Finally, Zhang et al. [36], in contrast with the results shown above, revealed that the AUC for the sole TyG index was 0.615, suggesting a weak predictive ability for AF incidence in NAFLD patients. However, when they combined TyG with traditional risk factors, the predictive value for AF was significantly improved, as evidenced by an AUC of 0.857 (*P* = 0.001). The sensitivity and specificity of the TyG model were 0.68 and 0.72, respectively. The TyG model had a higher predictive power for AF than traditional risk factors alone.

## Discussion

In this study, a systematic review and meta-analysis of 50,921 individuals in nine studies were performed (Table 1) to explore the association between TyG and the incidence of AF. We performed also a subgroup analysis: (1) post-ablation, (2) post-procedure, and (3) AF versus non-AF populations. The highlighted findings of the current study are: (1) AF incidence is higher in the group with high TyG levels compared to the lower TyG group; (2) the TyG index is higher in AF patients, compared to the normal population. AF is associated with higher morbidity and mortality rates due to related complications such as heart failure, cardiomyopathies, and thromboembolic events [37]. Despite various management options for AF, the prevalence of AF is rising due to new diagnostic methods. Therefore, recognition of AF risk factors and predictors may help clinicians in order to identify the patients that have a higher risk of AF, helping early detection of AF. In this context, several are the biomarkers identified and tested for risk stratification of AF, including the C-reactive protein (CRP) [38], the fibroblast growth factor-23 (FGF-23) [39], the high sensitivity troponin I [40], Galectin-3 [41], the N-terminal pro-B-type natriuretic peptide (NT-proBNP) [38, 39, 42–44], and several micro-RNAs [45]. However, other biomarkers associated with baseline risk factors, such as diabetes [46] and IR [47], have also been considered due to their bilateral diagnostic and prognostic usage.

Among the tools to measure IR in clinical practice, the TyG index has been established to be one of the easier-to-measure and cost‐effective parameters with a diagnostic and prognostic value comparable to other IR markers. In this regard, previous studies have correlated this surrogate marker of IR with several disorders, including COVID-19 [48], cerebrovascular disease or ischemic stroke [21, 49], hypertension [50], metabolic dysfunction-associated fatty liver disease [51], NAFLD [52], heart failure [18], acute myocardial infarction [53], and diabetes. In addition, our meta-analysis demonstrated that in the total AF population, the TyG index has a higher predictive value (SMD 1.23, 95% CI 0.71 to 1.75). Moreover, as shown in our subgroup analysis, there is no statistical difference between AF and non-AF groups (SMD 1.45, 95% CI -0.41 to 3.31). Conversely, in post-ablation (SMD 1.25, 95% CI 1.07 to 1.43) and post-procedure patients (SMD 0.99, 95% CI 0.78 to 1.20), AF was associated with a higher TyG index than their control groups.

The clinical utility of our findings includes considering TyG as an easy-to-use marker of IR in patients with AF. Considering the limitations of other common markers of IR, such as HOMA-IR, such as complexity of measurement or higher cost of measurement, TyG can be a valuable and reliable biomarker of IR [54]. Even in some studies, the TyG index outperformed HOMA-IR in evaluating IR, which makes it a potentially more useful index in the AF population as well [55, 56]. Our findings suggest an association between the TyG index and AF, however, the prognostic impact of TyG on outcomes of AF has not been investigated in these included studies. Meanwhile, the presence of DM and glycemic dysregulation in patients with AF has been shown to increase the risk of adverse outcomes such as cardiovascular mortality, sudden cardiac death, and stroke [57]. All of this highlights the need for further studies aiming at assessing this role.

## Strengths and limitations

This study was the first to assess the role of the TyG index in AF with a systematic review and meta-analysis. By pooling the data from these analyses, we found a higher TyG index in the subjects with AF than those without AF. Assessment of several outcomes and providing an insight into diagnostic ability are among the strengths of this study. This study will pave the way for future studies to specifically assess IR in AF pathology and prognosis through measurement of the TyG index. However, there are five main limitations to be disclosed. First, a high heterogeneity was observed in the analysis. Although performing subgroup analysis reduced the overall heterogeneity, these differences might stem from those in clinical settings and designs in each study. So, our study could be interpreted as a demonstrator of a possible relationship between the TyG index and AF, emphasizing the need for further larger studies. The second limitation is the number of included studies in the analyses which were low and limited our findings as well as some of the methods used such as publication bias assessment by funnel plots, Egger’s test, and meta-regression which is mainly performed in cases of higher than ten studies [58]. Third, different AF definitions could be a source of bias in our study, in addition to different follow-up durations for assessment of incident AF could be another limiting factor of our study. Fourth, different cut-off values across the included studies for the TyG index, may result in differences in categorizing individuals into the low or high TyG value groups. Fifth, the observational nature of the included studies prevents us from drawing any causal relationship conclusion. Hence, further larger studies investigating the association between the TyG index, and this high-risk population are warranted.

## Conclusion

Based on this study’s findings, the TyG index can be used as an IR marker for AF. Clinicians can take advantage of this index in patients with AF as well as its recurrence after ablation and other high-risk procedures. Further studies and association with other factors primarily influencing IR lifestyle modifications, pharmacological interventions, and other confounding variables (e.g., comorbidities) are needed to confirm the findings of the current study.

### Supplementary Information

**Additional file 1: Table S1.** Search strategy for each database. **Table S2.** Qualities of included studies based on NOS. **Table S3.** Multivariate meta-regression for meta-analysis of TyG index in patients with AF vs. healthy controls. **Figure S1.** Sensitivity analysis by leave-one-out method for meta-analysis of TyG levels in patients with AF and controls. **Figure S2.** Funnel plot by trim-and-fill method for meta-analysis of TyG levels in patients with AF and controls. **Figure S3.** Bubble plot for meta-regression of the mean age in the meta-analysis of TyG levels in patients with AF and controls. **Figure S4.** Bubble plot for meta-regression of the publication year in the meta-analysis of TyG levels in patients with AF and controls. **Figure S5.** Bubble plot for meta-regression of the sample size in the meta-analysis of TyG levels in patients with AF and controls. **Figure S6.** Bubble plot for meta-regression of the male percentage in the meta-analysis of TyG levels in patients with AF and controls. **Figure S7.** Forest plot for meta-analysis of TyG index in patients with and without AF using mean difference.

## Data Availability

The datasets used and/or analyzed during the current study are available from the corresponding author upon reasonable request.
