# Supplementary material for: Assessing the association between triglyceride-glucose index and atrial fibrillation: a systematic review and meta-analysis
Source: Eur J Med Res. 2024 Feb 12;29:118. doi: 10.1186/s40001-024-01716-8 (PMC10860290; doi:10.1186/s40001-024-01716-8)
Supplement: Supplementary file 1 — Additional file 1: Table S1. Search strategy for each database. Table S2. Qualities of included studies based on NOS. Table S3. Multivariate meta-regression for meta-analysis of TyG index in patients with AF vs. healthy controls. Figure S1. Sensitivity analysis by leave-one-out method for meta-analysis of TyG levels in patients with AF and controls. Figure S2. Funnel plot by trim-and-fill method for meta-analysis of TyG levels in patients with AF and controls. Figure S3. Bubble plot for meta-regression of the mean age in the meta-analysis of TyG levels in patients with AF and controls. Figure S4. Bubble plot for meta-regression of the publication year in the meta-analysis of TyG levels in patients with AF and controls. Figure S5. Bubble plot for meta-regression of the sample size in the meta-analysis of TyG levels in patients with AF and controls. Figure S6. Bubble plot for meta-regression of the male percentage in the meta-analysis of TyG levels in patients with AF and controls. Figure S7. Forest plot for meta-analysis of TyG index in patients with and without AF using mean difference. [file 40001_2024_1716_MOESM1_ESM.docx]

**Additional Materials**

***Table S1.*** *Search strategy for each database*

| **Query** | | **Results**  **(August 22, 2023)** |
| --- | --- | --- |
| ***PubMed*** | | |
| #1 | (“Atrial Fibrillation” OR “Atrial Fibrillation”[MeSH] OR “Atrial Fibrillations” OR “Fibrillation, Atrial” OR “Auricular Fibrillation” OR "Fibrillation, Auricular" OR "Persistent Atrial Fibrillation" OR “Atrial Fibrillation, Persistent” OR "Atrial Fibrillations, Persistent" OR "Fibrillation, Persistent Atrial" OR "Fibrillations, Persistent Atrial" OR "Persistent Atrial Fibrillations" OR "Familial Atrial Fibrillation" OR "Atrial Fibrillation, Familial" OR "Atrial Fibrillations, Familial" OR "Familial Atrial Fibrillations" OR "Fibrillation, Familial Atrial" OR "Fibrillations, Familial Atrial" OR "Paroxysmal Atrial Fibrillation" OR "Atrial Fibrillation, Paroxysmal" OR "Atrial Fibrillations, Paroxysmal" OR "Fibrillation, Paroxysmal Atrial" OR "Fibrillations, Paroxysmal Atrial" OR "Paroxysmal Atrial Fibrillations") | 107,980 |
| #2 | (“lipid ind*” OR “TyG” OR “triglyceride glucose” OR “triglyceride-glucose” OR “triglyceride-glucose index”) | 24,659 |
| **#3** | **#1 AND #2** | **69** |
| ***SCOPUS*** | | |
| #1 | TITLE-ABS-KEY(“Atrial Fibrillation” OR “Atrial Fibrillations” OR “Fibrillation, Atrial” OR “Auricular Fibrillation” OR "Fibrillation, Auricular" OR "Persistent Atrial Fibrillation" OR “Atrial Fibrillation, Persistent” OR "Atrial Fibrillations, Persistent" OR "Fibrillation, Persistent Atrial" OR "Fibrillations, Persistent Atrial" OR "Persistent Atrial Fibrillations" OR "Familial Atrial Fibrillation" OR "Atrial Fibrillation, Familial" OR "Atrial Fibrillations, Familial" OR "Familial Atrial Fibrillations" OR "Fibrillation, Familial Atrial" OR "Fibrillations, Familial Atrial" OR "Paroxysmal Atrial Fibrillation" OR "Atrial Fibrillation, Paroxysmal" OR "Atrial Fibrillations, Paroxysmal" OR "Fibrillation, Paroxysmal Atrial" OR "Fibrillations, Paroxysmal Atrial" OR "Paroxysmal Atrial Fibrillations") | 149,509 |
| #2 | TITLE-ABS-KEY(“lipid ind*” OR “TyG” OR “triglyceride glucose” OR “triglyceride-glucose” OR “triglyceride-glucose index”) | 5,985 |
| **#3** | **#1 AND #2** | **28** |
| ***Embase*** | | |
| #1 | (“Atrial Fibrillation” OR “Atrial Fibrillations” OR “Fibrillation, Atrial” OR “Auricular Fibrillation” OR "Fibrillation, Auricular" OR "Persistent Atrial Fibrillation" OR “Atrial Fibrillation, Persistent” OR "Atrial Fibrillations, Persistent" OR "Fibrillation, Persistent Atrial" OR "Fibrillations, Persistent Atrial" OR "Persistent Atrial Fibrillations" OR "Familial Atrial Fibrillation" OR "Atrial Fibrillation, Familial" OR "Atrial Fibrillations, Familial" OR "Familial Atrial Fibrillations" OR "Fibrillation, Familial Atrial" OR "Fibrillations, Familial Atrial" OR "Paroxysmal Atrial Fibrillation" OR "Atrial Fibrillation, Paroxysmal" OR "Atrial Fibrillations, Paroxysmal" OR "Fibrillation, Paroxysmal Atrial" OR "Fibrillations, Paroxysmal Atrial" OR "Paroxysmal Atrial Fibrillations") | 229,188 |
| #2 | (“lipid ind*” OR “TyG” OR “triglyceride glucose” OR “triglyceride-glucose” OR “triglyceride-glucose index”) | 26,428 |
| **#3** | **#1 AND #2** | **91** |
| ***Web Of Science*** | | |
| #1 | TS=(“Atrial Fibrillation” OR “Atrial Fibrillations” OR “Fibrillation, Atrial” OR “Auricular Fibrillation” OR "Fibrillation, Auricular" OR "Persistent Atrial Fibrillation" OR “Atrial Fibrillation, Persistent” OR "Atrial Fibrillations, Persistent" OR "Fibrillation, Persistent Atrial" OR "Fibrillations, Persistent Atrial" OR "Persistent Atrial Fibrillations" OR "Familial Atrial Fibrillation" OR "Atrial Fibrillation, Familial" OR "Atrial Fibrillations, Familial" OR "Familial Atrial Fibrillations" OR "Fibrillation, Familial Atrial" OR "Fibrillations, Familial Atrial" OR "Paroxysmal Atrial Fibrillation" OR "Atrial Fibrillation, Paroxysmal" OR "Atrial Fibrillations, Paroxysmal" OR "Fibrillation, Paroxysmal Atrial" OR "Fibrillations, Paroxysmal Atrial" OR "Paroxysmal Atrial Fibrillations") | 119,668 |
| #2 | TS=(“lipid ind*” OR “TyG” OR “triglyceride glucose” OR “triglyceride-glucose” OR “triglyceride-glucose index”) | 5,226 |
| **#3** | **#1 AND #2** | **15** |

Total: 203

After removing duplicates: 123

***Table S2.*** *Qualities of included studies based on NOS*

| **Study** | **Selection** | | | | **Comparability** | **Outcome** | | | **Overall score** |
| --- | --- | --- | --- | --- | --- | --- | --- | --- | --- |
|  | **Representation of exposed cohort** | **Selection of the non-exposed cohort** | **Ascertainment of exposure** | **Outcome of interest presence** | **-** | **Assessment of outcome** | **Sufficient length of follow-up** | **Loss to follow-up** |  |
| **Muhammad et al. (2022)** | * | * | * | * | * | - | * | * | 7 |
| **Tang et al. (2022)** | * | * | * | * | * | * | * | * | 8 |
| **Chen et al. (2022)** | * | - | * | * | * | * | * | * | 7 |
| **Shi et al. (2022)** | * | * | * | * | * | * | * | * | 8 |
| **Liu et al. (2023)** | * | * | * | * | * | * | * | * | 8 |
| **Ling et al. (2022)** | * | * | * | * | * | * | - | * | 8 |
| **Zhang et al. (2023)** | * | * | * | * | * | - | * | * | 7 |
| **Zhang et al. (2022)** | * | * | * | * | * | - | * | * | 7 |
| **Wei et al. (2021)** | * | * | * | * | * | * | * | * | 8 |

***Table S3.*** *Multivariate meta-regression for meta-analysis of TyG index in patients with AF vs. healthy controls*

| **Moderator** | **No. of studies** | **Slope** | **95% CI** | **p-value** |
| --- | --- | --- | --- | --- |
| **Publication year** | 6 | 0.7270 | 0.0458 to 1.4083 | 0.036 |
| **Sample size** | 6 | -0.0006 | -0.0010 to -0.0002 | 0.003 |
| **Mean age** | 6 | 0.1250 | -0.1394 to 0.3895 | 0.389 |
| **Male %** | 6 | -0.0708 | -0.1653 to 0.0237 | 0.142 |

***
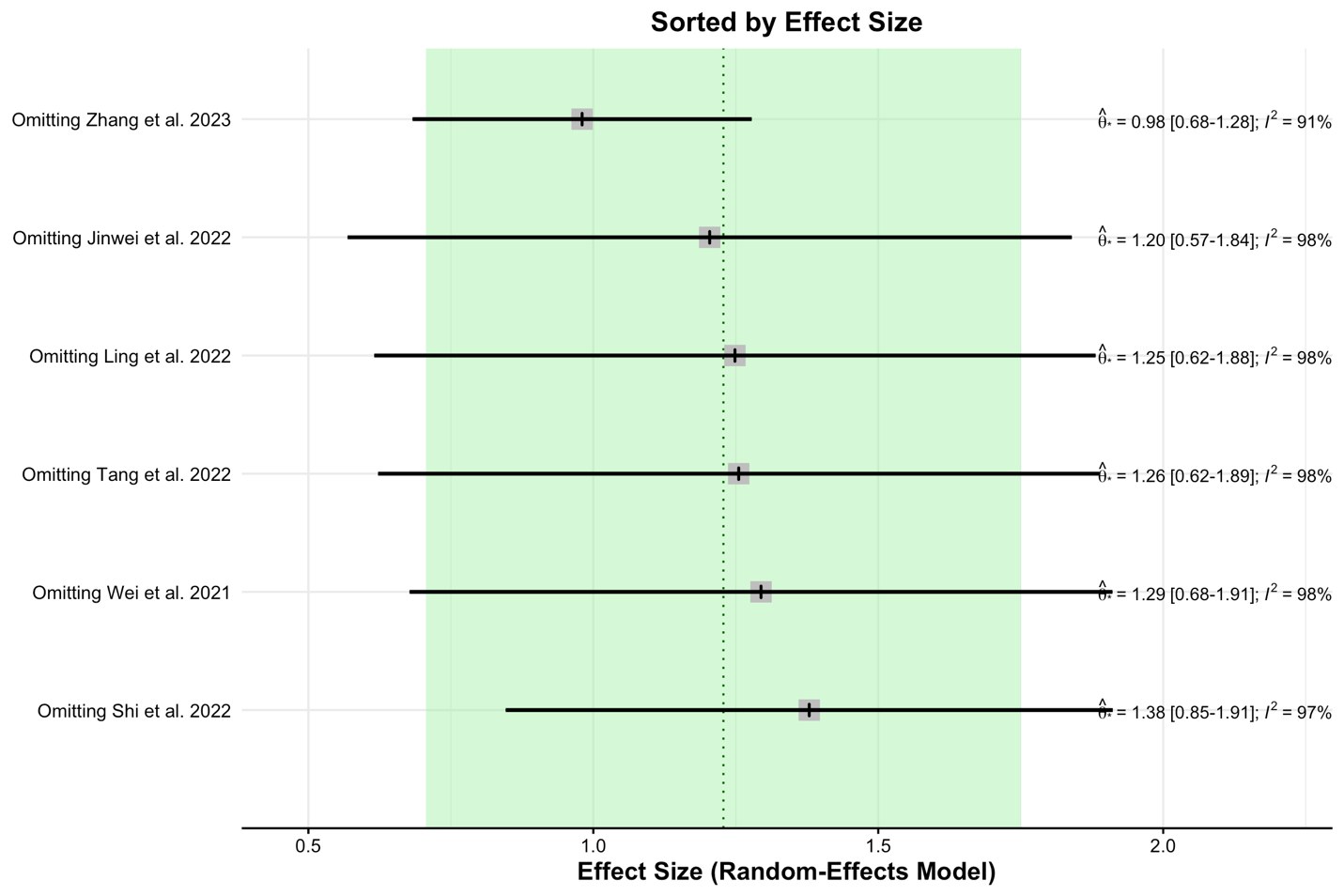
***

***Figure S1.*** *Sensitivity analysis by leave-one-out method for meta-analysis of TyG levels in patients with AF and controls*

*
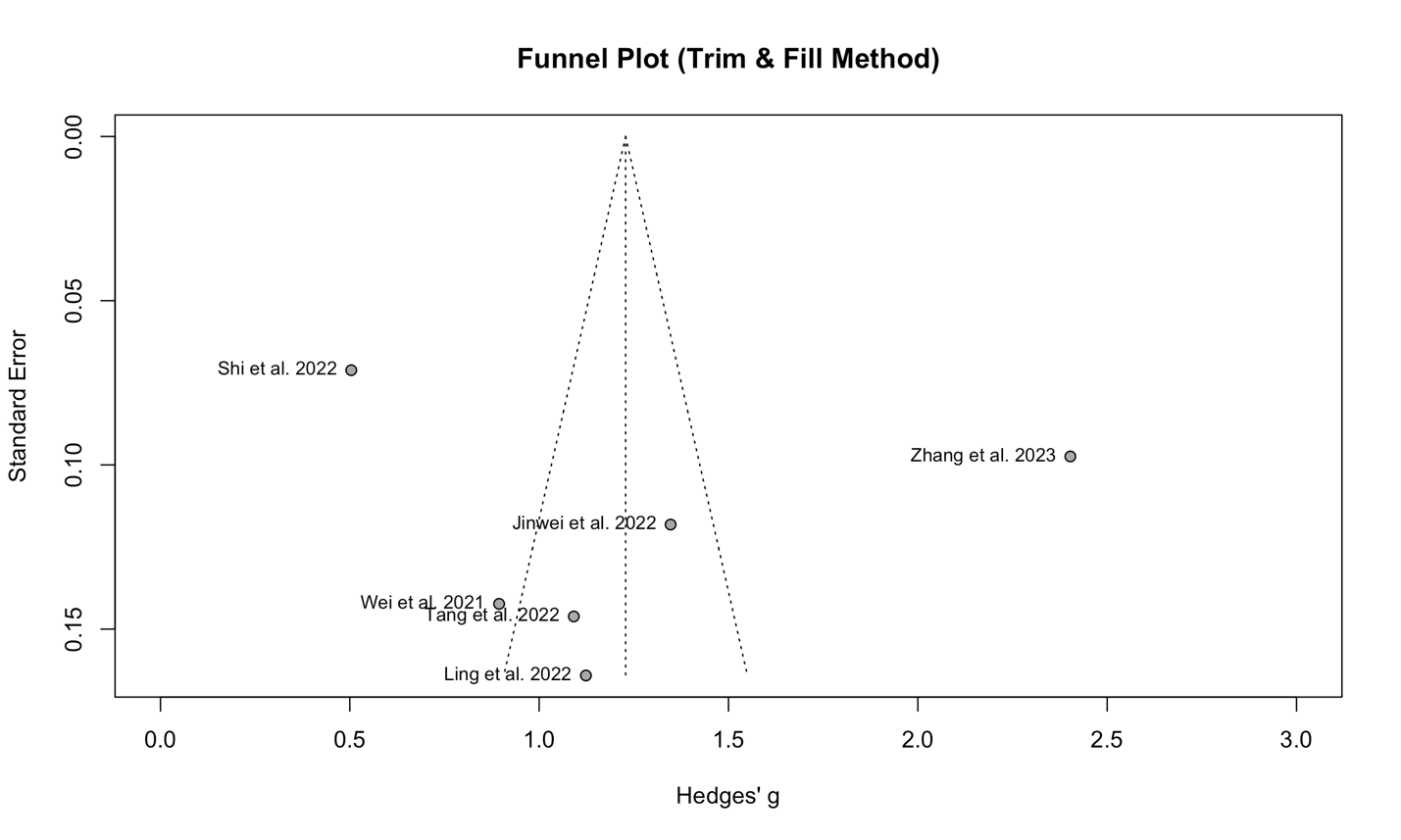
*

***Figure S2.*** *Funnel plot by trim-and-fill method for meta-analysis of TyG levels in patients with AF and controls*

*
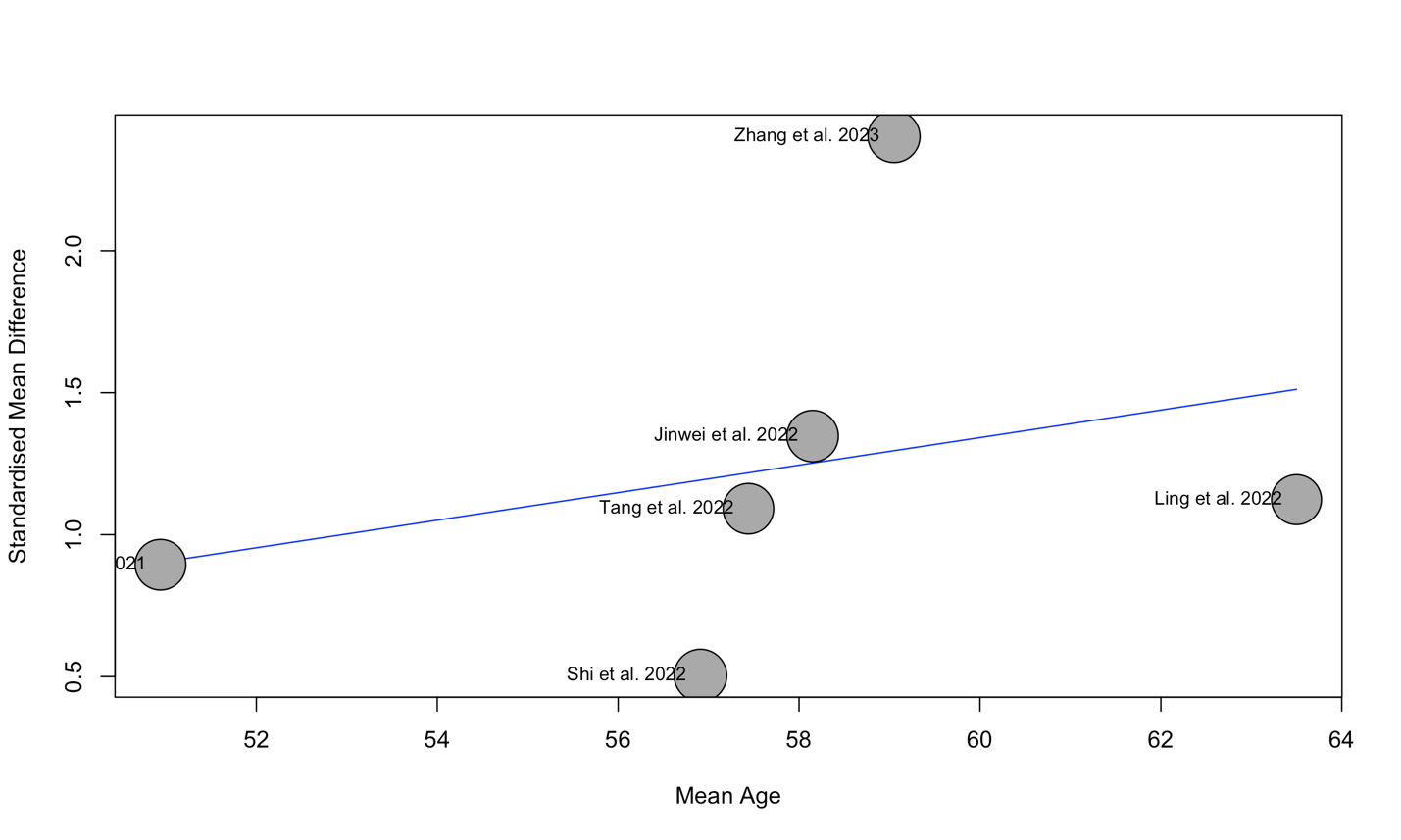
*

***Figure S3.*** *Bubble plot for meta-regression of the mean age in the meta-analysis of TyG levels in patients with AF and controls*

*
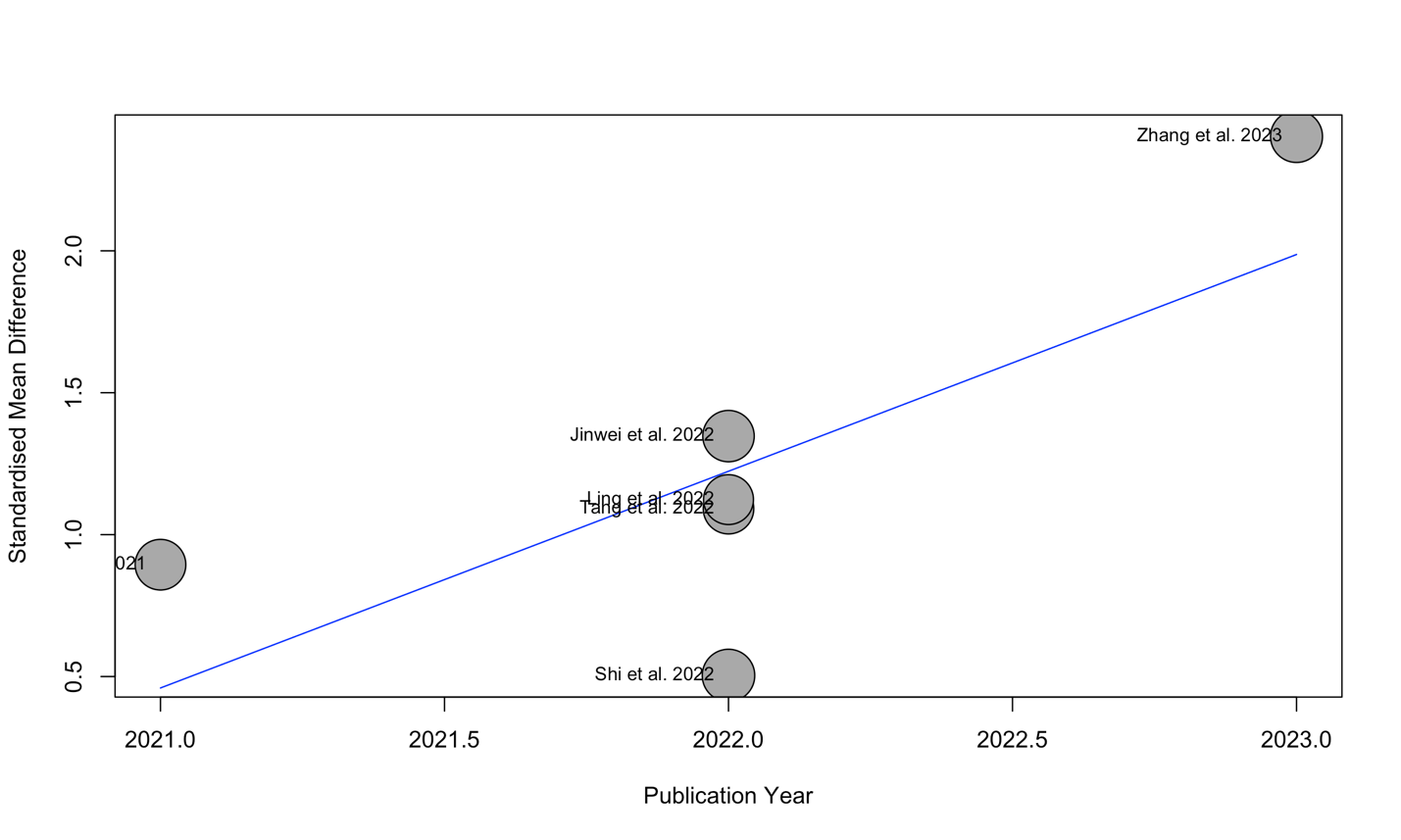
*

***Figure S4.*** *Bubble plot for meta-regression of the publication year in the meta-analysis of TyG levels in patients with AF and controls*

*
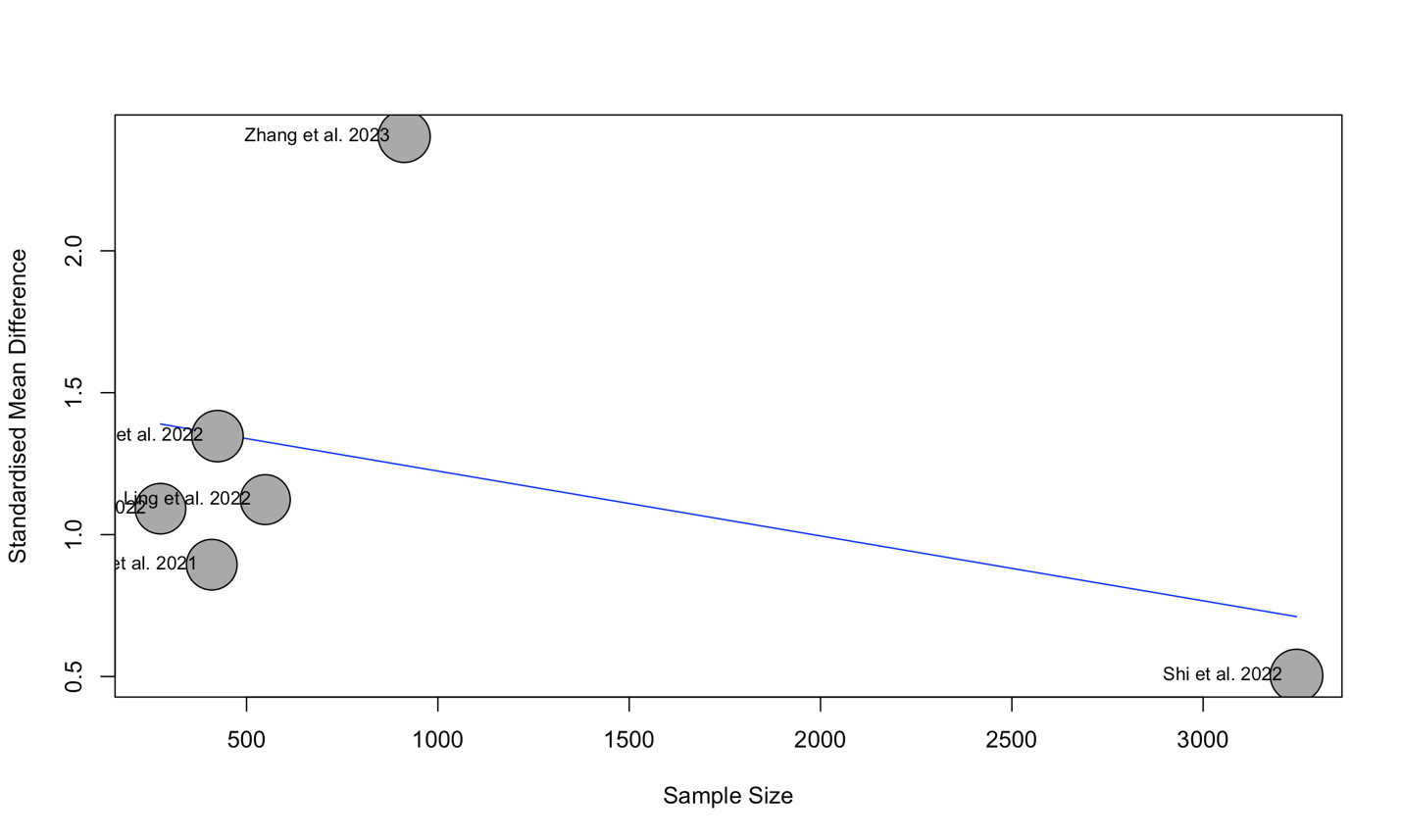
*

***Figure S5.*** *Bubble plot for meta-regression of the sample size in the meta-analysis of TyG levels in patients with AF and controls*

*
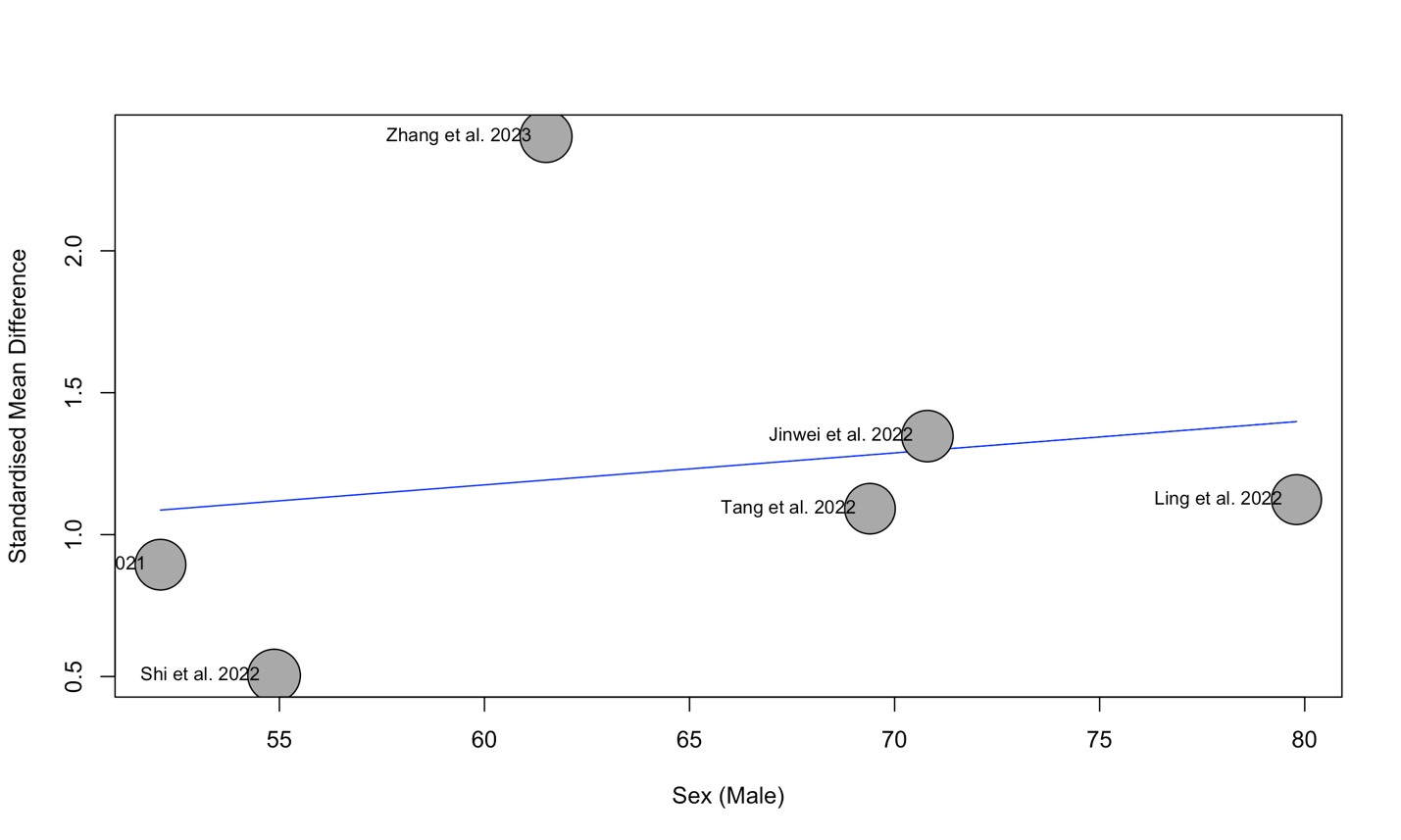
*

***Figure S6.*** *Bubble plot for meta-regression of the male percentage in the meta-analysis of TyG levels in patients with AF and controls*

*
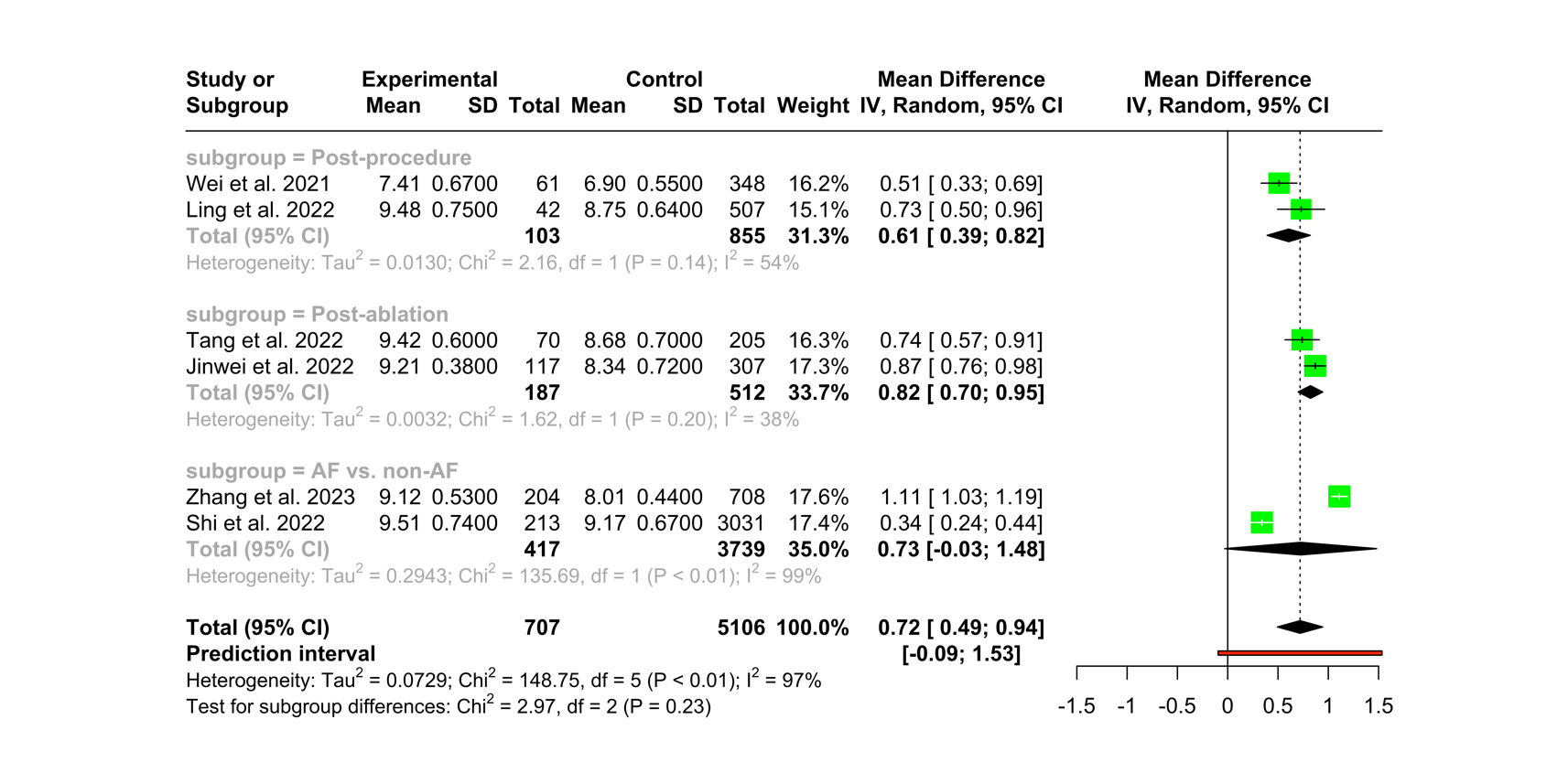
*

***Figure S7.*** *Forest plot for meta-analysis of TyG index in patients with and without AF using mean difference*
